# Hemoglobin level, degree of mobilization, and pneumonia are associated with the development of postoperative delirium in cemented hemiarthroplasty after femoral neck fracture

**DOI:** 10.1007/s00068-024-02613-9

**Published:** 2024-08-07

**Authors:** Julia Riemenschneider, Pascal Dobrawa, Ramona Sturm, Simon L. Meier, René Verboket, Ingo Marzi, Philipp Störmann

**Affiliations:** Department of Trauma Surgery and Orthopedics, University Hospital Frankfurt, Goethe University, Theodor-Stern-Kai 7, D-60590 Frankfurt/Main, Germany

**Keywords:** Postoperative delirium, Proximal femoral neck fracture, Hip hemiarthroplasty, Complications

## Abstract

**Purpose:**

The aim of this retrospective study was to identify independent prognostic factors for developing a postoperative delirium (POD) in patients suffering from a proximal femoral neck fracture and treated by implantation of a hemiprosthesis.

**Methods:**

A retrospective study, including patients with hip hemiarthroplasty due to a femoral neck fracture between 2011 and 2020 was performed. Demographic data, preexisting conditions, intra-/postoperative complications, mobilization and laboratory results were extracted from the patients’ charts. The different parameters were analyzed comparing patients with and without POD.

**Results:**

412 patients, mean age of 81 ± 9.58 years were included, 66.5% (*n* = 274) were female, 18.2% (*n* = 75) of them developed a POD. Significantly higher incidence of POD was associated with older age (*p* < 0.001), lower level of haemoglobin (*p* < 0.001), higher post-surgery interleukin 6 (IL 6) level (*p* = 0.008), higher postoperative level of leukocytes (*p* = 0.01). Patients with POD received more units of packed red blood cells (PRBC) (*p* = 0.007). Patients with no mobility limitations pre-operatively developed POD less frequently (*p* = 0.01), whereas suffering from pneumonia (*p* = 0.03) or limited mobility postoperatively increased the risk of POD (*p* < 0.001).

**Conclusion:**

This study could help to identify patients with a risk for developing POD after a hemiarthroplasty in femoral neck fractures. As a consequence, frequent controls of Hb, IL 6 and leucocytes levels to avoid anemia and infections, as well as the well surgical treatment to guarantee a good postoperative outcome.

## Introduction

A femoral neck fracture is one of the most common fracture types [1]. An epidemiological data analysis performed by Rupp et al. between 2009 and 2019 showed an incidence of 120/100.000 persons per year in Germany. This incidence increases with higher age and is higher in the female population [1]. Due to the demographic change with an increasingly elderly population and the occurrence of osteoporosis which is more prevalent in this group and is considered as a predictive factor of femoral neck fractures, an increase in this particular injury pattern is expected [2–4].

It is known that a delayed operative treatment (> 24–48 h) of such fractures results in higher morbidity and mortality [5, 6]. Several treatment options exist with the aim of a rapid mobilization after surgery. The applied treatment type depends on the fracture type, fragment dislocation, and the patient’s individual circumstances such as age, comorbidities and fitness. In conclusion, several studies have recommended an osteosynthesis for younger, active patients and different types of hip replacement for older, less active patients with potential comorbidities such as osteoporosis. Non-surgical treatment is possible in a few exceptional cases [5–7]. For dislocated fractures in the elderly, hemiarthroplasty (HA) (with a high ratio with cemented stems) can be considered the standard procedure in surgical treatment [5, 7, 8]. Advantages of this technique are shorter operation times and on average a lower blood loss compared to Total Hip Arthroplasty (THA), and the potential for immediate postoperative mobilization with full weight-bearing [9, 10].

Although this is the standard of care, the complication rate is relatively high, not at least because of the often-numerous pre-existing conditions in these elderly patients. The most frequent of these complications are deep vein thrombosis (1%), wound infections (4%), general infections like pneumonia or infections of the urinary tract (6%) and dislocation of the joint (0,5–11%) [5, 11].

A further, less frequently observed problem in cases of surgically treated elderly is the development of a postoperative delirium (POD). POD has been reported in between 5% and 56% of the elderly population following surgery of any type [12–15]. The different types of POD are either hyperactive, hypoactive or a mixed form of usually reversible mental disorders, and these can be screened using different screening tools [16]. Several predictive factors such as infections, high blood loss, electrolyte derailment and inadequate depth of narcosis have been discussed [17–19]. Delayed diagnosis of POD, as well as incorrect treatment increases the 1-year-mortality postoperatively, results in prolonged hospital stays, and might lead to persistent cognitive deficits [20, 21].

Since POD is often observed in patients requiring surgery due to femoral neck fractures and might worsen the outcome [22], the purpose of this retrospective study was to evaluate independent predictive factors that might cause a POD following cemented hemiarthroplasty after femoral neck fractures in the elderly.

## Materials and methods

A retrospective study at our level-1-trauma centre was performed. Patients suffering from a femoral neck fracture who were treated with cemented hemiarthroplasty (Zimmer, Freiburg, Germany, MS30 stem, modular bipolar femoral head) between 01/2011 and 12/2020 were included. Patients were identified using the International Statistical Classification of Diseases and Related Health Problems Version 10 (ICD-10) codes of the German Diagnosis Related Groups (G-DRG). Femoral neck fractures were identified by the ICD Code S72.0. In order to obtain a comparable patient population, all patients who underwent different surgical treatments other than cemented hemiarthroplasty (e.g., total hip replacement) were then excluded. All patients received general anesthesia for the procedures. All patient characteristics and disease-specific aspects were extracted from the patient records.

Patients with POD were compared to patients without POD. Patient charts were used for data collection. The diagnosis was made after checking a patient’s orientation to time, location, person, and situation, whether he or she had any circadian rhythm disruption and whether there was an attention deficit disorder after excluding other organic reasons (e.g. stroke or dehydration) based on the ICDSC [23] a score of ≥ 4 points was counted as POD. This procedure is repeated at least twice a day and documented in the patient’s chart. If there were deficits in several categories, the delirium was diagnosed by the surgical resident at the ward. A short scoring system developed by Belleli et al. which is nowadays used in intermediate and in intensive care units to assess these categories [24].

Values are reported as the mean ± standard deviation (SD) for continuous variables and as percentages for categorical variables. All analyses were performed using the Statistical Package for Social Sciences (SPSS for Mac) version 27.0 (IBM Inc., Chicago, IL, USA). Demographic and clinical parameters were evaluated using bivariate analysis. At first normality tests were performed. P-values for categorical variables were derived from Chi-square or two-sided Fisher’s exact test and for continuous variables from Student’s t-test or the Mann-Whitney U-test. A p-value < 0.05 was considered statistically significant. To identify risk factors independently associated with the development of POD, a stepwise logistic regression model was utilized, and risk factors from the bivariate analysis with a p-value < 0.05 were included in the model.

Written approval from the institutional review board and ethics committee of the Goethe University medical faculty (EV 2022 − 641) was obtained 3/2022. Because of the retrospective character of this study the ethics committee of the Goethe University in Frankfurt waived off the need for informed consent. This study was performed in accordance with relevant guidelines and regulations. Data were collected afterwards until 12/2022. This study followed the STROBE guidelines for observational studies (Strengthening the Reporting of Observational Studies in Epidemiology) and the RECORD guidelines (Reporting of studies Conducted using Observational Routinely collected Data) [25, 26].

## Results

A total of 412 patients met the inclusion criteria. The mean age was 81.3 ± 9.6 years, and 66.5% (*n* = 274) of the patients were female. Overall, 18.2% (*n* = 75) developed a POD.

Patients suffering from POD were significantly older (POD: 84.8 ± 8.6 yrs. vs. no POD: 80.6 ± 9.6 yrs., *p* < 0.001, OR (95% CI) = 1.06 (1.03–1.09)), while male sex did not significantly influence the development of POD (*p* = 0.69) (Table 1).

**Table 1:** Results of the general conditions and the influence of developing a postoperative delirium (POD) with a significant association for a higher age

	Total Mean (SD)	No POD Mean (SD)	POD Mean (SD)	*p*-value
age (y)	81.3 (9.6)	80.6 (9.6)	84.8 (8.6)	< 0.001
time to surgery (h)	37.0 (64.0)	37.6 (67.8)	34.0 (43.7)	0.67

SD = standard deviation, y = years, h = hours

Patients without mobility limitations before trauma were less likely to develop a POD than patients with such limitations (*p* = 0.01). The different kinds of mobility limitation (use of wheelchair, crutches, walker or total immobilization) did not significantly contribute to develop a delirium. Similarly, we could not demonstrate any difference with regard to the type of trauma or the use of anticoagulation medication (Table 2).

**Table 2:** Results of gender, mobilization before trauma, type of trauma and the presence of permanent anticoagulation. Free mobility pre-operatively results in lower rates of postoperative delirium (POD)

	Total % (*n*)	No POD % (*n*)	POD % (*n*)	*p*-value
male sex	33.5 (138)	32.9 (111)	36.0 (27)	0.69
immobile	0.7 (3)	0.9 (3)	0.0 (0)	1.00
wheel chair	3.9 (16)	4.7 (16)	0.0 (0)	0.09
walker/crutches	17.7 (73)	16.3 (55)	24.0 (18)	0.13
free	45.9 (189)	49.0 (165)	32.0 (24)	0.01
low energy trauma	93.0 (383)	92.0 (310)	97.3 (73)	0.13
high energy trauma	1.0 (4)	1.2 (4)	0 (0)	1.00
pathological Fractures	6.1 (25)	6.5 (22)	4.0 (3)	0.59
anticoagulation	19.2 (79)	18.7 (63)	21.3 (16)	0.63
platelet aggr. Inhib.	31.8 (131)	31.8 (107)	32.0 (24)	1.00

In chronic pre-existing conditions, the presence of heart failure and chronic dialysis showed a tendency for an increased risk of POD, although without reaching statistical significance (*p* = 0.13 and 0.14, respectively). When considering general complications during the postoperative course, only the occurrence of pneumonia showed a significant association with the occurrence of POD (*p* = 0.03). Deranged electrolytes, both pre- and postoperatively, tended to increase the incidence of POD, but also without statistical significance (pre *p* = 0.12, post *p* = 0.14) (Table 3).

**Table 3:** Results of the pre-existing conditions and acute health events during or after surgical treatment and the influence of developing a postoperative delirium (POD). A significant influence could only be seen in patients with pneumonia

	Total % (*n*)	No POD % (*n*)	POD % (*n*)	*p*-value
CHD/HI	17.7 (73)	16.3 (55)	24.0 (18)	0.13
COPD	6.3 (26)	6.2 (21)	6.7 (5)	0.80
renal insufficiency	15.8 (65)	16.3 (55)	13.3 (10)	0.60
dialysis	4.0 (14)	2.7 (9)	5.7 (5)	0.14
art. Hypertension	60.0 (247)	59.6 (201)	61.3 (46)	0.90
diabetes I/II	19.9 (82)	20.8 (70)	16 (12)	0.43
CNS related diseases	26.2 (108)	26.7 (90)	24.0 (18)	0.67
cardiac event	10.9 (45)	10.1 (34)	14.7 (11)	0.30
acute renal insufficiency	7.0 (29)	6.2 (21)	10.7 (8)	0.21
pneumonia	10.2 (42)	8.6 (29)	17.3 (13)	0.03
urinary tract infection	14.8 (61)	14.8 (50)	14.7 (11)	1.00
electrolyte derailment preop.	59.5 (245)	57.6 (194)	68.0 (51)	0.12
electrolyte derailment postop.	96.8 (399)	96.1 (324)	100.0 (75)	0.14

CHD = coronary heart disease, CI = cardiac insufficiency, COPD = chronical obstructive pulmonary disease, CNS = central nerve system

Patients who could not be mobilized to at least standing postoperatively were significantly more likely to have POD (best mobilization bedside/chair; no POD: 72.3% vs. POD: 27.7%, *p* < 0.001) (Table 4).

**Table 4:** Results of the postoperative mobilization and the influence of developing a postoperative delirium (POD). A limited mobilization postoperatively increased the risk to suffer from POD

	Total % (*n*)	No POD % (*n*)	POD % (*n*)	*p*-value
free with delay	17.7 (73)	18.5 (62)	14.7 (11)	0.51
bedside/chair	33.3 (137)	29.5 (99)	50.7 (38)	< 0.001
walker/crutches	15.5 (64)	14.9 (50)	18.7 (14)	0.48

Regarding routine laboratory parameters that were analyzed, patients with POD showed a significantly lower lowest postoperative haemoglobin (Hb) (POD: 7.43 ± 1 g/dl vs. 8 ± 1.3 g/dl, *p* < 0.001) and a higher rate of transfusion of packed red blood cells (PRBCs) (POD 3.7 ± 4 units vs. no POD 2.7 ± 4 units, *p* = 0.007). Furthermore, the first postoperative interleukin-6 (Il 6) value was significantly higher (POD 188.0 ± 159.4 pg/ml vs. no POD 131.8 ± 135.0 pg/ml), *p* = 0.008), as was the highest postoperative leukocyte count (POD: 16.9 (± 9.0)/nl, no POD 14.0 (± 6.3)/nl, *p* = 0.003). Further laboratory parameters are given in Table 5.

**Table 5:** Low level of hemoglobin (Hb) and high levels of Interleukin 6 (IL 6) and leukocytes postoperative are associated with postoperative delirium (POD). Laboratory parameters given in the table were available for all patients (n = 412)

	Total Mean (SD)	No POD Mean (SD)	POD Mean (SD)	p-value
hemoglobin on admission (g/dl)	12.2 (1.9)	12.2 (1.9)	12.4 (1.9)	0.54
hemoglobin post-surgery (g/dl)	10.1 (1.5)	10.1 (1.5)	9.93 (1.4)	0.27
hemoglobin lowest level (g/dl)	7.9 (1.2)	8.0 (1.3)	7.43 (1.0)	< 0.001
sodium on admission (mmol/l)	138.2 (4.7)	138.1 (4.6)	138.9 (4.5)	0.17
sodium post-surgery (mmol/l)	139.3 (4.0)	139.1 (3.8)	140.3 (4.6)	0.06
potassium on admission (mmol/l)	4.2 (0.6)	4.2 (0.6)	4.3 (0.8)	0.61
potassium post-surgery (mmol/l)	4.2 (0.4)	4.2 (0.4)	4.2 (0.4)	0.99
calcium on admission (mmol/l)	2.3 (0.2)	2.3 (0.2)	2.2 (0.2)	0.44
calcium post-surgery (mmol/l)	2.0 (0.1)	2.0 (0.1)	2.0 (0.1)	0.99
CRP on admission (mg/dl)	2.7 (4.4)	2.7 (4.3)	3.0 (5.0)	0.60
CRP post-surgery(mg/dl)	7.4 (5.2)	7.5 (5.2)	6.7 (5.5)	0.52
CRP highest level post-surgery(mg/dl)	12.5 (7.4)	12.8 (7.3)	11.4 (7.6)	0.16
IL-6 post-surgery (pg/ml)	144.8 (142.4)	131.8 (135.0)	188.0 (159.4)	0.008
IL-6 highest level post-surgery (pg/ml)	667.1 (3347.1)	558.9 (3533.3)	1031.6 (2651.0)	0.33
leukocytes on admission (/nl)	11.0 (4.9)	10.9 (4.6)	11.5 (6.3)	0.31
leukocytes post-surgery (/nl)	12.2 (5.1)	12.2 (4.9)	12.5 (5.9)	0.66
leukocytes highest level post-surgery (/nl)	14.5 (7.0)	14.0 (6.3)	16.9 (9.0)	0.01
creatinine pre-surgery (mg/dl)	1.2 (0.8)	1.4 (0.8)	1.3 (1.0)	0.23
creatinine post-surgery (mg/dl)	1.0 (0.8)	1.0 (0.8)	1.1 (1.0)	0.23
packed red blood cells (PRBC)	2.5 (4.0)	2.3 (4.0)	3.7 (3.7)	0.007
platelet concentrates	0.2 (1.5)	0.25 (1.6)	0.1 (0.5)	0.46

SD = standard deviation, CRP = C-reactive protein

Logistic regression showed that older age (*p* < 0.001, OR (95% CI) 1.06 (1.03–1.09)) can be associated with the development of POD, among other factors. In addition, a higher postoperative Hb level appeared to be protective against the development of POD (*p* < 0.001, OR (95% CI) 0.65 (0.51–0.82)) and accordingly the administration of more PRBC was associated with an increased incidence (*p* = 0.01, OR (95% CI) 1.07 (1.02–1.13)) (Table 6).

**Table 6:** Logistic regression analysis. OR = odds ratio, CI = confidence interval

	*p*-Value	OR (95% CI)
age	< 0.001	1.06 (1.03–1.09)
free preoperative mobilization	0.01	2.04 (1.2–2.4)
pneumonia	0.06	2.23 (1.1–4.5)
postoperative mobilization into chair/bedside	0.01	2.04 (1.2–3.46)
higher hemoglobin level	< 0.001	0.65 (0.51–0.82)
IL6 level post surgery	0.01	1 (1.001–1.004)
leucocytes highest level	0.003	1.05 (1.02–1.09)
packed red blood cells (PRBCs)	0.01	1.07 (1.02–1.13)

## Discussion

This was a retrospective study of an elderly population with femoral neck fractures treated by hemiarthroplasty at the author’s institution investigating predictive factors associated with the development of POD. To the best of our knowledge, this is the first study showing that among other things a high level of IL-6 or leucocytes, the postoperative development of pneumonia, a low postoperative level of haemoglobin, a delayed mobilization and a better pre-operative activity status. are associated with POD.

Postoperative delirium is an underestimated complication. Ruchholtz et al. and other authors have reported a frequency of 5–56%, with the incidence of POD being higher in the elderly population [12, 13, 27]. In general, the first symptoms can occur 24–48 h after surgery [14, 28]. Regardless of the type of delirium– hyper-, hypoactive or mixed forms– it affects the outcome and compliance with treatment and is, among others, associated with persisting cognitive deficits [29, 30]. Our results are consistent with a previous study reporting 18.2% patients with POD after hemiarthroplasty in an elderly population, but due to the retrospective character of our study, we cannot make a statement in regard to long-term outcomes in our cohort. Recent studies by Schenning et al. demonstrated that specific operative treatments, like hip surgery, were associated with a higher prevalence of POD [14, 28]. Because of the fact that certain predisposing factors cannot be influenced, it is more important to identify risk factors that might be optimized pre- and postoperatively [14, 17].

Suffering from a femoral neck fracture has a tremendous impact on the quality of life and longevity for most elderly patients. Several treatment options for femoral neck fractures exist, but considering current guidelines and studies hemiarthroplasty with partly cemented stems is the actual standard for the elderly population [5, 10]. A recent study from the Norwegian Arthroplasty Register demonstrated an increase of the rate of cemented stems in hemiarthroplasty in elderly patients up to 91% [31]. Despite advances in care and increasing standardization of surgical therapy, these injuries boast a relatively high complication rate [5]. Common complications are wound infections, bleeding, and dislocation of the prosthesis [32–34]. As a result, surgical techniques as well as hygiene measures and antibiotic prophylaxis are regularly discussed with a view to further improving the postoperative outcome [5, 10, 33].

Among these general complications, POD is associated with higher disability and mortality after surgically treated femoral neck fractures. However, this often appears to be only a secondary consideration in attempts to improve treatment. In spite of following all guidelines, in particular the most timely surgical care possible, the 1-year mortality rate is still estimated between 20 and 30% [13, 34, 35]. Longer hospitalization and a poorer functional recovery of patients with POD have also been reported [29]. In a 5-year prospective follow-up study Lundström et al. demonstrated that 40% of all patients with POD in their cohort with femoral neck fracture developed a dementia and 72% died within 5 years [30]. There is a clear need for improvement in the management of POD to achieve a better outcome [36–38]. Bearing this in mind, we tried to identify independent risk factors that might result in POD.

Several studies have examined different potential risk factors for developing POD after hip fracture surgery, but there is no common consensus to date. Concerning age and medical comorbidities independent research show that a higher age > 80 years (which is in line with our results) arterial hypertension and COPD can contribute to the development of POD as individual risk factors. No clear associations with the development of POD have been found for other preexisting conditions, such as diabetes, kidney and heart disease. This is consistent with the results of our study, in which we were able to identify, for example pre-existing heart failure led to a slightly increased risk of POD, but without reaching statistical significance [29, 35, 39, 40]. Furthermore, the meta-analysis by Smith et al. showed that postoperative infections, such as pneumonia or urinary tract infections, can promote the occurrence of POD. These results are supported by our study, which showed a strong correlation between the occurrence of pneumonia and POD [39, 41]. In our group, we were unable to show any correlation between the presence of a pathological fracture and the occurrence of delirium. This is in contrast to the study by Zhao et al., for example, who describe pathological fractures as a risk factor. This question remains open for the time being, as there was only a very small number of pathological fractures in our group [42].

Rapid postoperative mobilization is associated with fewer postoperative complications. This applies especially for lower rates of pulmonary or urinary infections and even a lower rate of mortality after hip surgery, but there is no evidence for a lower incidences of POD [43–46]. No studies have tested the influence of the activity level before trauma on the development of a delirium to date. Nevertheless, our results indicate that patients who do not have any restrictions on mobility are less likely to develop POD, whereas postoperatively, patients who suffer a longer restriction of mobilization suffer from POD significantly more often. However, the data do not allow us to conclude whether restricted mobilization is causal for POD or whether it is a pure association. In summary, however, it can be said that good mobilization, both preoperatively and postoperatively, should be considered a central component of delirium prophylaxis.

The repeated monitoring of laboratory parameters is a postoperative standard in intermediate and intensive care units. All patients included to this study spent at least 24 h at one of those units referring to the post operative standard. Checking creatinine and electrolytes like sodium or potassium is done to help control patient’s fluid balance. Imbalances in these parameters are considered as a risk factor for developing POD. Besides this, abnormal levels of calcium, potassium or magnesium can cause certain types of dementia [47–49]. Furthermore, it is common to regularly check inflammatory markers like CRP and IL-6. Even though these markers are nonspecific, they are usually used as predictive factors for early postoperative infections, e.g. pneumonia [15, 50]. In our study, we found a correlation between POD after femoral neck surgery and high levels of postoperative IL-6 and leucocytes as well as the occurrence of pneumonia. It seems most likely to us that elevated IL-6 levels indicate the occurrence of infection, and that infection promotes the development of POD. Thus, an association rather than a causality has to be assumed here. If this is the case, increased IL-6 levels in the cohort described by us, could be an indicator of the need for POD prophylaxis. Besides the standard laboratory parameters several studies have examined other biomarkers and their effects on developing a POD e.g. independent studies have detected an increase in apolipoprotein E ε4 in patients with delirium [51, 52], which appears to be a promising approach in the detection of POD.

In this study we found that the level of haemoglobin was significantly lower in patients that developed POD. This observation is consistent with other studies of cohorts undergoing major vascular, orthopedic, or cardiac surgery. Almost 50% of patients suffers from a POD. In the case of an emergency operation the risk is even higher [53, 54]. The low level of haemoglobin often accompanies high blood loss in surgical patients, which is often considered as a predictive factor for delirium. Postoperative anaemia as a risk factor for POD has been discussed in previous studies [41]. While there is a recommendation of a restrictive haemoglobin transfusion trigger < 7 g/dl for certain critical patients [55], blood transfusions are considered as a risk factor for POD, creating a dilemma for patient blood management [35, 40, 56]. This is consistent with the results of our study– patients administered a higher number of PRBCs had a significantly higher risk of developing POD.

Nowadays strict indication guidelines for the transfusion of PRBCs exist, and blood transfusions are recommended in case of low haemoglobin levels. An indication will be given if the Hb level is < 7 g/dl (4.3 mmol/L) regardless of the ability for compensation and < 8 g/dl (5.0 mmol/dL) in case of risk factors like pre-existing cerebrovascular or cardiovascular pre-existing health problems or a clinical hypoxia [57]. With patient blood management in mind, it should nevertheless be considered whether the transfusion limits for our elderly clientele should be interpreted more generously in attempts to avoid POD. Respecting the low availability of PRBCs, the balance between need and benefit in elderly patients with proximal femoral fractures is important. We found that patients with a Hb level < 8 g/dl had a significant risk for developing POD; this might be considered an appropriate threshold in these patients.

In addition, a higher postoperative Hb level appears protective against the development of POD.

As mentioned above POD is one of the most common complications in elderly people, with an incidence of almost 60% [12, 13]. Different medical and non-pharmacological treatment options exist, but there is no consensus about prophylaxis for high-risk patients [36, 37]. The latest guideline about the treatment of delirium recommends the monitoring of the previously mentioned laboratory parameters and if necessary their correction [21]. However, other studies have discussed the positive effect of improving cognitive skills or visual and hearing adaptations as part of the “Hospital Elder Life Program” as a preventive strategy [36, 37, 58], as well as the allocation of a specific specially trained nurse as the individual contact person for high-risk patients [36]. To prevent POD many pre-, intra-, and postoperative factors can be optimized. In the case of POD after hemiarthroplasty in patients with proximal femoral fractures we found that haemoglobin level, post-operative IL-6 levels, and postoperative level of leukocytes could be useful for early identification of the development of POD. Predictive factors like age and mobility before and after surgery must be noted and respected, too, as part of delirium prophylaxis.

### Limitations

The general limitations of a retrospective study design need to be considered. The analyzed protocols were not standardized, and the data collected on pre- and postoperative mobility were partly incomplete. Because of the retrospective character of this study and regarding the comorbidities the single categorizations for example COPD or renal insufficiency are missing. Maybe the severity of those could influence the results differently, but for categorizing them correctly in many cases data are missing. Likewise, there were no general criteria concerning postoperative patients and the study only considered patients with the diagnosis of POD on the treatment and discharge documents. It was not possible to determine the exact time at which POD developed. In this context, it is known, that a stay on ICU is an independent risk factor for the development of POD [59]. All of our patients are admitted to ICU postoperatively, due to the retrospective data we were not able to give an exact length of ICU stay which might promote the development of POD. This should be analyzed in upcoming prospective studies. Moreover, the variance of the individual values differed especially for biomarkers and there were partly no preoperative results for IL6 because of different laboratory standards in the emergency room and our intermediate care unit. As a result, data were not available for every category for all patients. This might have affected individual results with higher standard deviations and lower significance. However, the large size of our cohort is a strength of this study, as well as the focus on only one disease and treatment method. The final limitation is that the available data cannot be used to imply an association with delirium or, for example, whether low Hb levels are causal for the development of POD.

## Conclusion

In this study of 412 patients with identical treatment of femoral neck fracture by cemented hemiarthroplasty, 18.2% developed POD. We were able to demonstrate independent risk factors for the development of POD in our collective. Among these, different parameters can be controlled, influenced and thus the prophylaxis of POD can be supported.

Taken together the most important findings are that a lower level of haemoglobin, higher levels of post-surgery interleukin 6 and leukocytes as well as suffering from pneumonia and a limited mobility postoperatively increased the risk of POD significantly. As a consequence, the aim in treating patients with femoral neck fractures with cemented hemiarthroplasty should be frequent controls of Hb, IL 6 and leucocytes levels to avoid anaemia and infections, as well as the well surgical treatment to guarantee a rapid mobilization under full weight bearing postoperative.

## Data Availability

No datasets were generated or analysed during the current study.
